# Effects of Pleiotrophin Overexpression on Mouse Skeletal Muscles in Normal Loading and in Actual and Simulated Microgravity

**DOI:** 10.1371/journal.pone.0072028

**Published:** 2013-08-28

**Authors:** Giulia Maria Camerino, Sabata Pierno, Antonella Liantonio, Michela De Bellis, Maria Cannone, Valeriana Sblendorio, Elena Conte, Antonietta Mele, Domenico Tricarico, Sara Tavella, Alessandra Ruggiu, Ranieri Cancedda, Yoshinobu Ohira, Daniela Danieli-Betto, Stefano Ciciliot, Elena Germinario, Dorianna Sandonà, Romeo Betto, Diana Conte Camerino, Jean-François Desaphy

**Affiliations:** 1 Section of Pharmacology, Department of Pharmacy & Drug Sciences, University of Bari – Aldo Moro, Bari, Italy; 2 Department of Oncology, Biology, and Genetics, University of Genova, Genova, Italy; 3 Graduate School of Medicine and Frontier Biosciences, Osaka University, Osaka, Japan; 4 Department of Biomedical Sciences, University of Padova, Padova, Italy; 5 Institute of Neuroscience, National Research Council, Padova, Italy; University of Iowa, United States of America

## Abstract

Pleiotrophin (PTN) is a widespread cytokine involved in bone formation, neurite outgrowth, and angiogenesis. In skeletal muscle, PTN is upregulated during myogenesis, post-synaptic induction, and regeneration after crushing, but little is known regarding its effects on muscle function. Here, we describe the effects of PTN on the slow-twitch soleus and fast-twitch *extensor digitorum longus* (EDL) muscles in mice over-expressing PTN under the control of a bone promoter. The mice were maintained in normal loading or disuse condition, induced by hindlimb unloading (HU) for 14 days. Effects of exposition to near-zero gravity during a 3-months spaceflight (SF) into the Mice Drawer System are also reported. In normal loading, PTN overexpression had no effect on muscle fiber cross-sectional area, but shifted soleus muscle toward a slower phenotype, as shown by an increased number of oxidative type 1 fibers, and increased gene expression of cytochrome c oxidase subunit IV and citrate synthase. The cytokine increased soleus and EDL capillary-to-fiber ratio. PTN overexpression did not prevent soleus muscle atrophy, slow-to-fast transition, and capillary regression induced by SF and HU. Nevertheless, PTN exerted various effects on sarcolemma ion channel expression/function and resting cytosolic Ca^2+^ concentration in soleus and EDL muscles, in normal loading and after HU. In conclusion, the results show very similar effects of HU and SF on mouse soleus muscle, including activation of specific gene programs. The EDL muscle is able to counterbalance this latter, probably by activating compensatory mechanisms. The numerous effects of PTN on muscle gene expression and functional parameters demonstrate the sensitivity of muscle fibers to the cytokine. Although little benefit was found in HU muscle disuse, PTN may emerge useful in various muscle diseases, because it exerts synergetic actions on muscle fibers and vessels, which could enforce oxidative metabolism and ameliorate muscle performance.

## Introduction

Pleiotrophin (PTN), also called heparin affin regulatory peptide (HARP), heparin binding-growth associated molecule (HB-GAM), or osteoblast stimulating factor-1 (OSF-1), is a heparin-binding cytokine expressed by several cell types during early differentiation and up-regulated after tissue injury [1]. It has been shown that PTN is specifically involved in bone formation [2], neurite outgrowth [3] and angiogenesis [4]. PTN is also expressed in skeletal muscle and upregulated during in vitro myogenesis and post-synaptic induction, as well as in rat soleus muscle during regeneration after crushing [5]–[7]. Nevertheless little is known regarding the effects of PTN on skeletal muscle.

Since PTN over-expression in transgenic mice is associated with an increased bone mass and mineralization and may protect from experimentally-induced osteoporosis [8]–[9], the mice drawer system (MDS) experiment was designed to accommodate PTN-overexpressing mice aboard the International Space Station (ISS) with the aim to verify whether PTN can also protect mice from space related osteoporosis [10]. In the MDS experiment, wild type and transgenic mice over-expressing PTN under the control of the human bone specific osteocalcin promoter were exposed to a near-zero gravity on board the ISS for a record-breaking period of three months. On return to Earth, analysis of bone microarchitecture in these mice revealed some protective effects of PTN against microgravity’s negative effects on weight-bearing bones, likely resulting from stimulation of osteoblast activity [11].

During the MDS experiment, an international muscle team participated to a tissue sharing program with the aim to investigate the effects of long-duration spaceflight (SF) on mouse skeletal muscles. Skeletal muscles, especially the postural ones, constitute a main target of microgravity environment. Microgravity exposure during SF can be considered as an extreme condition mimicking the effects of disuse on the musculoskeletal apparatus occurring in Earth physiopathological conditions such as aging, bed rest, or forced immobilization. Muscle disuse occurring in humans in these conditions induces muscle fiber atrophy and alteration of muscle function, which can cause serious medical problems. Similar effects are also observed in hindlimb muscles of rodents using the hindlimb unloading (HU) model [12]. Analysis of the slow-twitch soleus muscle and the fast-twitch *Extensor Digitorum Longus* (EDL) muscles of wild-type mice after the MDS mission merely confirmed results obtained in mice and rats after shorter SF and revealed additional information [13]. The postural soleus muscle underwent a severe atrophy likely due to over-activation of the ubiquitin-dependent proteasome, changes in gene and protein expression in accord with the shift toward a faster and less oxidative phenotype, and alteration in gene expression of specific proteins involved in muscle function, which altogether likely contribute to muscle impairment [13].

Another interesting issue of the MDS experiment regards the possible effects of PTN overexpression on soleus and EDL muscle properties in microgravity environment. Although PTN was overexpressed in bones under the control of a bone-specific promoter, one may expect some influence of this factor on skeletal muscle either indirectly, through the partial protective effect on weight-bearing bones, or directly through paracrine effects on neighboring muscles. Thus we sought to investigate whether overexpression of PTN may represent a useful countermeasure against disuse-induced muscle impairment.

In this study, we examined the effects of PTN overexpression on Sol and EDL muscle fiber properties of control mice housed in normal laboratory cages (ground) or after 14 days of hindlimb-unloading, a well-recognized model of simulated microgravity. The HU model allowed to obtain further information regarding the effects of PTN overexpression on muscle gene expression and function. We also examined muscle vascularization because PTN is widely recognized as a pro-angiogenic factor. Results obtained on wild-type and PTN mice from the MDS mission are also reported to illustrate possible effects of SF. The results of this study indicate that PTN overexpression exerts a number of transcriptional and post-transcriptional effects in mouse Sol and EDL muscles in normal loading and both disuse conditions.

## Materials and Methods

### Ethics Statement

During the Mice Drawer System (MDS) experiment (pre-flight, during the flight and postflight), handling of mice was in accordance with the principles expressed in the “Guide for the care and the use of laboratory animals” (Office of Science and Health Reports, NIH, Bethesda, USA). Approval of the MDS experiment was obtained by the American Institutional Animal Care and Use Committee (IACUC protocol nuFLT-09-070 - KSC), the Ethics Committee of the Animal Facility of the National Institute for Cancer Research (Genoa, Italy), and the Public Veterinary Health Department (Ministero del Lavoro, della Salute e delle Politiche Sociali prot. n°4347-09/03/2009-DGSA.P.).

The hindlimb unloading (HU) experiments were performed in accordance with the Italian Guidelines for the use of laboratory animals, which conforms with the European Union Directive for the protection of experimental animals (2011/63/EU), and received approval from the Italian Health Department.

### Pleiotrophin-overexpressing Transgenic Mice

Transgenic mice from the BDF strain over-expressing PTN under the control of the human bone specific osteocalcin promoter show a faster bone formation process and an increased bone mineral density, as well as a decreased bone tissue atrophy in ovariectomized mice 8]. The original transgenic mice with the BDF genetic background were backcrossed in the C57BlJ10 strain to obtain a strain of PTN mice with a C57BlJ10 genetic background, because C57Bl mice better tolerated the MDS housing conditions in preliminary experiments [10].

### The Mice Drawer System Spaceflight Mission

The MDS spaceflight mission organized by the Italian Space Agency has been previously described in details [10]. Briefly, the MDS payload (Thales Alenia Space Italia, Vimodrone, Italy), was used to house 6 mice (3 wild-type C57BL mice and 3 transgenic mice overexpressing PTN) aboard the Space Shuttle and the International Space Station for a record-breaking, 91 days-long, space journey. The mice were 2-months old at the time of launch, and 5 months-old upon return to Earth. One WT and 2 transgenic mice survived to the mission, showing a normal behavior throughout the experiment and appearing in good health conditions at landing, as previously detailed [10]. Less than two hours after landing, the mice were sacrificed by CO_2_ inhalation and tissues were removed according to an international tissue sharing program. Hindlimb skeletal muscles, including Soleus (Sol) and *Extensor Digitorum Longus* (EDL) muscles, were promptly removed after death and frozen in liquid nitrogen for further analysis. Age-matched control WT (n = 3) and PTN (n = 3) mice were housed on ground in normal laboratory cages. Results obtained from the analysis of WT mouse skeletal muscles have been previously published [13]. In the present paper, we report the results obtained from the analysis of the two spaceflown PTN mice to evaluate the effects of PTN on actual microgravity. It is worth mentioning that the results from the MDS mission needs to be considered as preliminary, due to the limited number of available mice.

### Hindlimb Unloading Experiments

Wild-Type (Charles River Laboratories, Calco, Italy) and transgenic PTN C57Bl mice, aged 5–6 months, were housed individually in appropriate cages in an environmentally controlled room. The animals were randomly assigned to control (ground) and hindlimb unloaded (HU) experimental groups as follows: (1) WT ground mice (WT-ground, n = 15); (2) 14-days hindlimb-unloaded WT mice (WT-HU, n = 17); (3) ground transgenic mice overexpressing pleiotrophin (PTN-ground, n = 13); (4) 14-days hindlimb-unloaded transgenic mice overexpressing PTN (PTN-HU, n = 12). All mice had water *ad libitum* and received 8 g a day of standard rodent chow (Charles River, 4RF21). To induce muscle unloading, the animals of HU group were suspended individually in special cages for 2 weeks as described 14]. A thin string was linked at one extremity to the tail by sticking plaster and at the other extremity to the top of the cage. The length of the string was adjusted to allow the animals moving freely on the forelimbs, while the body was inclined at 30–40° from the horizontal plane. At the end of suspension, the mice were unfastened from the string and deeply anesthetized by intraperitoneal injection of urethane (1.2 g/kg body weight) to allow removing of Sol and EDL muscles. The same procedure was applied to WT and PTN ground mice maintained free in individual cages for 14 days. Muscles were used immediately for the functional experiments or frozen in liquid nitrogen and stored at −80°C for other studies. After surgery, animals were euthanized by an overdose of urethane.

### Cross Sectional Area (CSA) Analysis

Muscles were frozen in liquid nitrogen in a slightly stretched position. Serial cross sections (8-µm thick) were cut in a cryostat microtome set at −24±2°C (Slee Pearson, UK). To measure the cross-sectional area (CSA) of individual fibers, muscle cryostat sections were stained for laminin, a major component of the basal lamina. Digital photographs were taken of each muscle section and the CSA was automatically measured as the internal laminin-unstained area by the ImageJ software (NIH, freeware imaging software) 13]. The semi-automatic method we use allows to measure most of the fibers in the cryostat section. Three cryostat sections were analyzed for each muscle sample. In total, the number of counted fibers ranged between 450 and 700 fibers per muscle.

### Fiber Typing and MyHC Isoform Content

Fiber typing was determined by immunofluorescence using combinations of the following monoclonal antibodies: BA-D5 that recognizes type 1 MHC isoform; SC-71 for type 2A MHC isoform; BF-F3, for type 2B MyHC isoform 15]. To detect the primary antibodies the following secondary antibodies were used: DyLight405 labeled goat anti mouse IgG, Fcc 2b subclass specific (115-475-207), to specifically detect BA-D5, DyLight488 labeled goat anti mouse IgG, Fcc 1 subclass specific (115-485-205), for SC71, and DyLight549 labeled goat anti mouse IgM (115-505-075), used to specifically detect BF-F3. Secondary antibodies were purchased from Jackson Immunoresearch, anti-HA, 16B12, from Covance, USA, and anti-myc, 9E10, from Roche. Pictures were collected with an epifluorescence DM5000 B microscope equipped with a DFC 300 FX digital camera (Leica, Germany). Single-color images were merged with Adobe Photoshop CS2 (Adobe Systems Inc.) to obtain a whole muscle reconstruction 13]. Images were also obtained in presence of the sole secondary antibodies to verify the absence of background fluorescence in our experiments (see Figure S1).

Analysis of MyHC isoforms was also performed by SDS-PAGE using the method of Talmadge and Roy [16], as previously described [13]. Shortly, small muscle fragments from Sol and EDL muscles were weighed, ground with a ceramic pestle in liquid nitrogen, and extracted at 2 mg/ml in SDS-PAGE sample buffer (62.5 mM Tris, pH 6.8, 2.3% SDS, 5% 2-mercaptoethanol, 10% glycerol). Forty mg of muscle sample was run on 8% SDS-PAGE slab gels. MyHC protein bands from whole muscles were revealed by Coomassie brilliant blue staining. MyHC isoform percentage composition was determined by densitometry of gels by using a Bio-Rad Imaging Densitometer (GS-670).

### Muscle Capillarization Analysis

Frozen 8 µm transverse cross-sections were cut in the mid-belly of the Sol and EDL muscles. Capillaries were visualized after a brief fixation in cold acetone followed by staining for alkaline phosphatase activity by incubation with 5-bromo-4-chloro-3-indolyl phosphate/nitro blue tetrazolium (FAST BCIP/NBT, Sigma-Aldrich, Milano, Italy) for 45 min at 37°C. Pictures were acquired using a DM RXA microscope (Leica, Mannheim, Germany) equipped with a high resolution Digital Eclipse DXM 1200 camera (Nikon, Sesto Fiorentino, Italy) (kindly provided by prof. Antonio Frigeri, University of Bari). The capillary-to-fiber ratio (C/F) were determined by counting capillaries and myofibres on three-four cryosections from each muscle, using the ImageJ freeware (NIH). The number of muscle fibers analyzed per muscle sample ranged from 530 to 870. The data are presented as mean ± SEM from n mice.

### Resting ion Conductance of Sarcolemma

Ion channel resting conductance were measured using the 2-intracellular microelectrodes technique 14,17]. EDL and Sol muscles were dissected from wild-type and PTN transgenic animals, immediately placed in a muscle containing bath immersed in normal or chloride-free physiological solution maintained at 30°C and perfused with 95% O_2_/5% CO_2_. The normal physiological solution contained (in mM): NaCl 148, KCl 4.5, CaCl_2_ 2.0, MgCl_2_ 1.0, NaHCO_3_ 12.0, NaH_2_PO_4_ 0.44, glucose 5.5, and pH 7.2. The chloride-free solution was prepared by equimolar substitution of methylsulfate salts for NaCl and KCl and nitrate salts for CaCl_2_ and MgCl_2_. The cable parameters of myofiber sarcolemma were determined from the electrotonic potentials elicited by square wave hyperpolarizing current pulse of 100-ms duration, using two intracellular microelectrodes in current-clamp mode. The membrane conductance is calculated from the values of input resistance, space constants and time constant and assuming a myoplasmic resistivity of 140 Ω·cm. The mean chloride conductance (gCl) is calculated as the mean total membrane conductance (gm) measured in normal physiological solution minus the mean potassium conductance gK measured in chloride-free solution.

### Fluorescence Measurements of Resting Intracellular Ca^2+^ Concentration

Fluorescence measurements were performed on small bundles of five to ten fibers lengthwise dissected from mice EDL and Sol muscles, as described elsewhere 14,18]. The muscle fibers were incubated with the fluorescent calcium probe fura-2 for 45–60 min at 22°C in physiological solution containing 5 µM of the acetoxymethyl ester (AM) form of the dye mixed to 10% (v/v) Pluronic F-127 (Molecular Probes, Leiden, The Netherlands). A QuantiCell 900 integrated imaging system (VisiTech International Ltd, UK) was used to acquire pairs of background-subtracted images of the fura-2 fluorescence emission (510 nm) excited at 340 and 380 nm. The equation used to transform fluorescence ratio in Ca^2+^]i values was Ca^2+^]i = (R-Rmin)/(Rmax-R)·K_D_·β, where R is the ratio of fluorescence excited at 340 nm to that excited at 380 nm; K_D_ = 145 nM; β, Rmin and Rmax were determined in situ in ionomycin-permeabilized muscle fibers. Because calibration parameters of FURA-2 can depend on muscle type and experimental condition [18], we determined the parameters of Grynkiewicz’s equation in each muscle examined for accurate calculation of calcium concentration.

### Quantitative Real-time PCR Analysis

Gene expression was measured by real time PCR in Sol and EDL muscles promptly frozen in liquid nitrogen after dissection and stored at −80°C. All the RT-PCR experiments were performed in agreement with the MIQE guidelines for qPCR, as published [19]. For each Sol and EDL muscle sample belonging to the HU mice group, total RNA was isolated using RNeasy Fibrous Tissue Mini Kit (Quiagen C.N. 74704) and quantified by using a spectrophotometer (ND-1000 NanoDrop, Thermo Scientific). Reverse transcription was performed on 400 ng of total RNA from each sample supplemented with 1 µl dNTP mix containing 10 mM of each dNTP (Roche N.C. 11277049001) and 1 µl Random Hexamers (50 µM) (Life Technologies C.N. n808-0127). After incubation at 65°C for 5 min, the mixture was completed with 4 µl First Standard Buffer (5X) (Life Technologies C.N. Y02321), 2 µl DTT (0,1 M) (Life Technologies C.N. Y00147), and 1 µl Recombinant RNasin® Ribonuclease Inhibitor (40 U/µl) (Promega C.N. N2511). After incubation at 42°C for 2 min, 1 µl Super Script II Reverse Transcriptase (200 U/µl) (Life Technologies C.N. 18064-014) was added to each sample. Sequential incubations were then performed at 25°C for 10 min, 42°C for 50 min. and 70°C for 15 min. For each Sol and EDL muscle sample belonging to the SF mice group, total RNA extraction was performed using the Qiagen RNeasy MicroKit (Quiagen), while reverse transcription and amplification was performed using Ovation Pico kit (NuGEN), as described previously [13].

Real-time PCR was performed in triplicate using the Applied Biosystems Real-time PCR 7500 Fast system, MicroAmp Fast Optical 96-Well Reaction Plate (Life Technologies C.N. 4346906), and MicroAmp Optical Adhesive Film (Life Technologies C.N. 4311971). Each reaction was carried out as singleplex reaction, to avoid that two pairs of primers can compete for Taq polymerase enzyme. TaqMan hydrolysis assay was performed using specific primer and probe sequences designed by ourselves or obtained from Applied Biosystems with specific assay IDs (see Table S1). The setup of reactions consisted in 8 ng of cDNA, 0,5 µl of TaqMan hydrolysis primer and probe set for commercial primers (TaqMan Gene Expression Assays, Life Technologies) or 360 pM of primers plus 200 pM of probe designed by us, 5 µl of TaqMan Universal PCR master mix (No AmpErase UNG 2x; Life Technologies C.N. 4324018), and Nuclease-free water not DEPC-treated (Sigma-Aldrich C.N. VC00021) for a final volume of 10 µl. The PCR conditions were as follows: step 1: 95°C for 20 s; step 2: 95°C for 3 s; and step 3: 60°C for 30 s; steps 2 and 3 were repeated 40 times. The results were compared with the relative gene standard curve obtained from 5 data points of 1∶4 serial dilutions. The mRNA expression of the genes was normalized to the housekeeping hypoxantine guanine phosphoribosyl transferase (HPRT1) gene. In previous studies, the HPRT1 appeared as the most stable gene in HU and SF conditions, as compared to β-actin and β2-microglobulin [13], [14].

### Statistical Analysis

Average data are presented as mean ± SEM from n mice. Mice from SF and HU experiments were age-matched, being 5–6 months old at the moment of muscle extraction. Control data (ground) were thus obtained indistinctly from mice housed in normal laboratory cages from the SF and/or HU experiments. Comparison of means between two experimental conditions was done using unpaired Student’s *t*-test. A P value minor to 0.05 was considered statistically significant.

## Results

### Effects of PTN Overexpression on Muscle CSA of Ground, HU, and SF Mice

**Figure 1:**
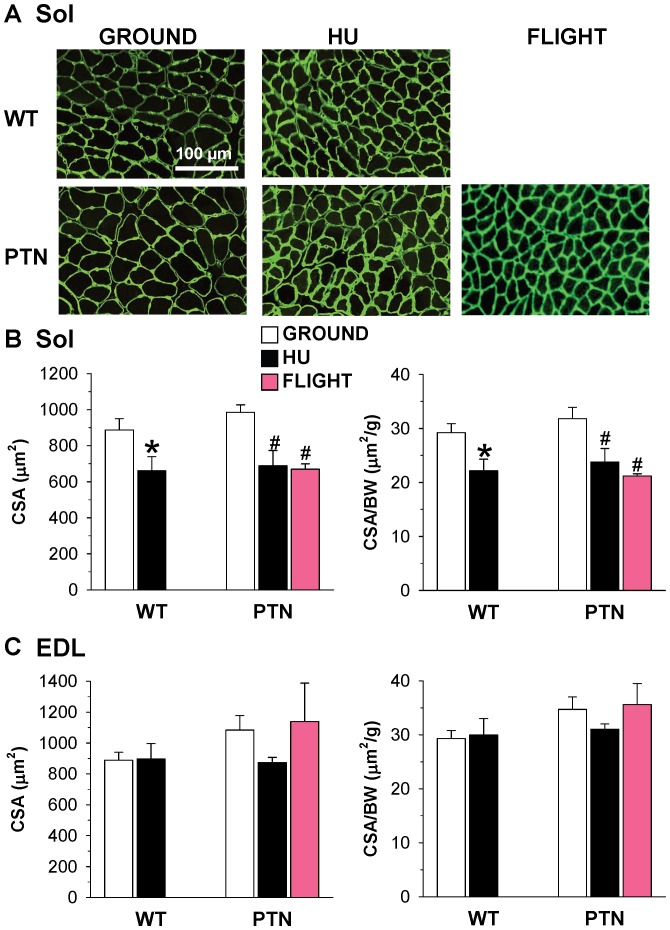
Muscle fiber size of wild-type and PTN-overexpressing mice after 14 days HU or 91 days space flight. A) representative Sol muscle cryosections stained with antibodies specific for laminin. The area inside the laminin staining was utilized to measure muscle fiber CSA. B) Effect of HU and spaceflight on CSA of Sol fibers and CSA-to-body weight ratio. C) Effect of HU and spaceflight on CSA of EDL fibers and CSA-to-body weight ratio. Each bar is the mean ± SEM from 2 to 9 mice. Statistical analysis, performed with two-tailed unpaired Student’s *t* test, indicates significant atrophy in soleus muscle of PTN mice after HU or spaceflight (* indicates *P*<0.05 versus WT-ground mice, and # indicates *P*<0.05 versus PTN-ground mice).

To evaluate the effects of PTN overexpression on muscle fiber growth and atrophy induced by actual and simulated microgravity, the CSA of Sol and EDL muscle fibers was measured on laminin-stained muscle (**Fig. 1A**). As expected, the Sol muscles of WT mice show a 25% reduction of CSA after HU (**Fig. 1B**). PTN had no significant effect on muscle CSA in normal loading, and atrophy of Sol muscle was still observed in PTN mice either after HU, with a 30% significant reduction of CSA, or after SF with a 32% CSA reduction. The CSA-to-body-weight ratio show the same trend (**Fig. 1B**). Thus, 14 days HU and 91 days SF produced comparable Sol muscle atrophy, and PTN overexpression was unable to protect Sol muscle against disuse atrophy. In contrast, no significant change in CSA and CSA-to-body weight ratio was observed in the EDL muscle of WT and PTN mice, confirming lack of atrophy in this muscle (**Fig. 1C**).

### Effects of PTN Overexpression on Muscle Phenotype in ground, HU, and SF Mice

**Figure 2:**
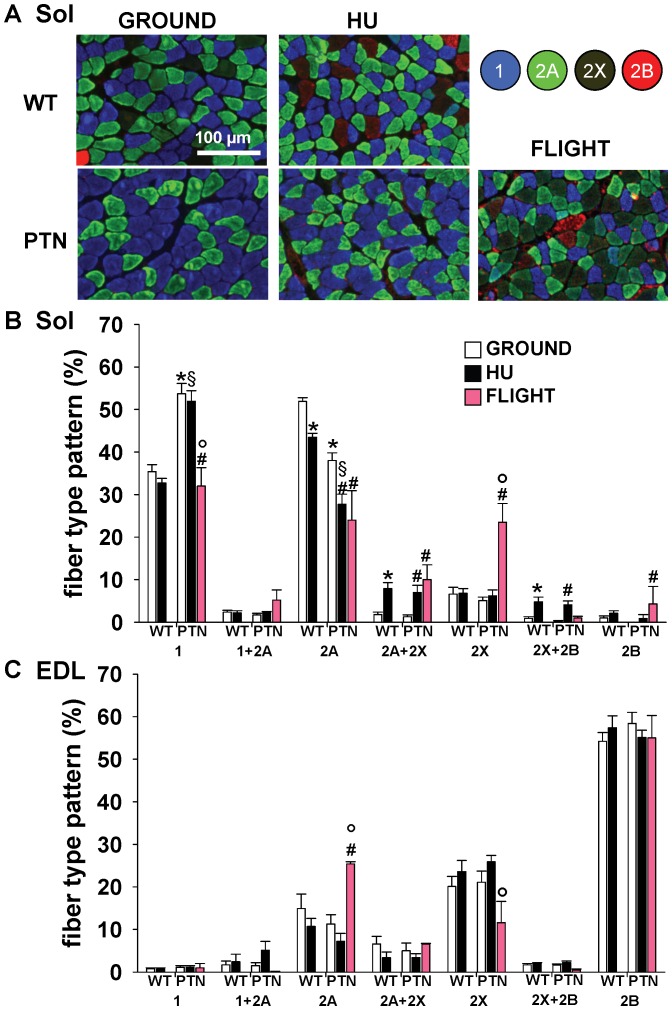
Muscle fiber typing of wild-type and PTN-overexpressing mice after 14 days HU or 91 days space flight. A) Representative soleus muscle sections immunostained by means of monoclonal antibodies specific for the different myosin heavy chain isoforms. B–C) Fiber type composition of Sol (B) and EDL (C) muscles determined as shown in A. Each bar is the mean ± SEM from 2-to-10 mice. Statistical analysis performed with unpaired Student’s *t* test indicates significant change (at least *P*<0.05) versus WT-G (*), WT-HU (§), PTN-G (#), and PTN-HU (°).

To determine the fiber type compositions of EDL and Sol muscles, serial muscle cryosections were stained with monoclonal antibodies specific for each MyHC subtype (**Fig. 2A**). As expected for mice, the Sol muscle appeared as a mixed muscle containing 35% of pure type 1 fibers, i.e. containing the slow MyHC-1 subtype, 52% of fast type 2A fibers, containing MyHC-2A, 7% of type 2X fibers and less than 1% of type 2B fibers (**Fig. 2B**). A small number of fibers co-expressed two MyHC subtypes. Unexpectedly, over-expression of PTN significantly affected the fiber type composition of Sol muscle, increasing the proportion of pure type 1 fibers to 54%, while reducing the proportion of pure type 2A fibers to 38% and pure 2X to 5%, whereas type 2B fibers were absent (**Fig. 2B**). This observation suggests that PTN transgene shifted Sol muscle toward a slower phenotype.

**Figure 3:**
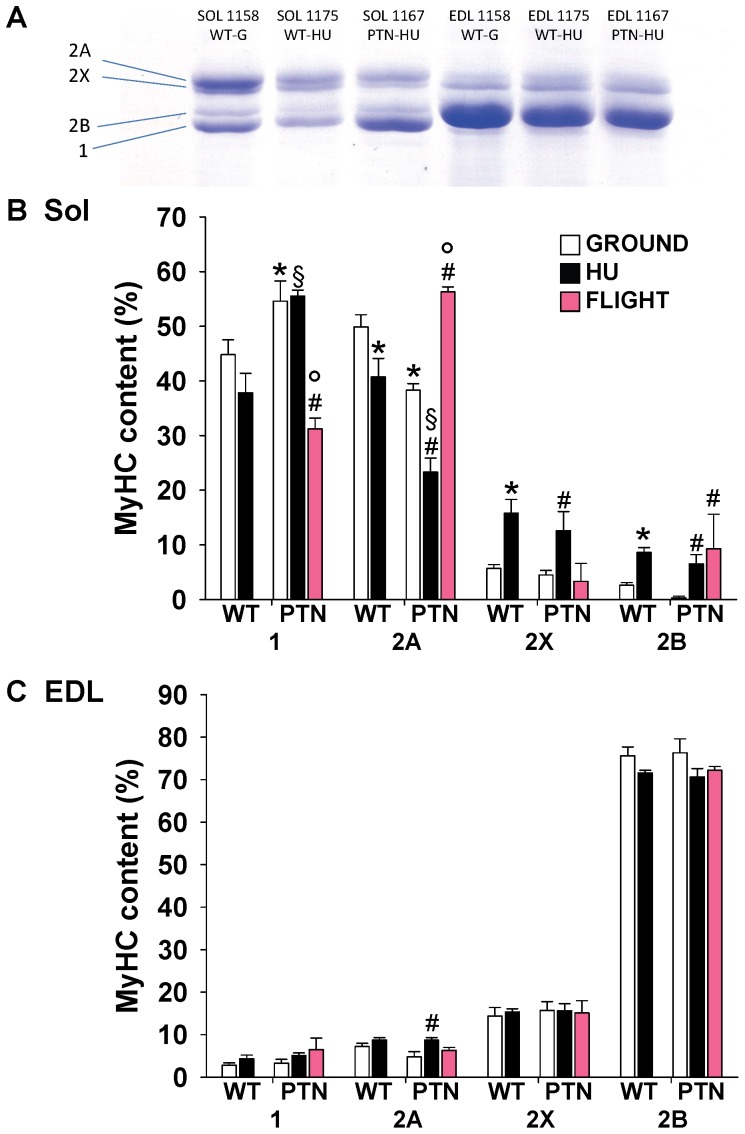
Muscle MyHC isoform composition of wild-type and PTN-overexpressing mice after 14 days HU or 91 days space flight. A) MyHC isoforms were separated by SDS-PAGE and stained with Coomassie blue. Densitometric analysis on MyHC isoforms is reported for Sol (B) and EDL (C) muscles. Each bar is the mean ± SEM from 2-to-7 mice. Statistical analysis performed with unpaired Student’s *t* test indicates significant change (at least *P*<0.05) versus WT-G (*), WT-HU (§), PTN-G (#), PTN-HU (°).

As expected, HU induced changes in MyHC expression pattern in WT mice toward a faster phenotype, with a reduction of type 2A fibers and the increase of hybrid type 2A–2X and type 2X-2B fibers. A similar slow-to-fast transition was observed after HU in PTN mice (**Fig. 2B**). As previously observed in WT mice [13], SF induced a slow-to-fast shift in Sol muscles of PTN mice by decreasing the proportion of type 1 and 2A fibers and increasing 2X and 2B fibers (**Fig. 2B**). Such an observation suggests that PTN overexpression was not able to prevent the disuse-induced phenotypic change in fiber type composition. The electrophoretic SDS-PAGE analysis of MyHC subtypes expression in the whole Sol muscle confirmed the results obtained on immunostained cryosections with 1) a shift of Sol muscle toward a slower phenotype in PTN mice on ground, and 2) a slow-to-fast phenotype shift induced by HU and SF in PTN mice (**Fig. 3A**).

The EDL muscle is an archetypical fast-twitch muscle, which express more than 70% of pure type 2B fibers and about 20% of type 2X fibers (**Fig. 2C**). In contrast to Sol muscle, the PTN transgene had no effect on the fiber type composition in the EDL. No substantial effect of HU or SF was observed in the EDL muscle of WT and PTN mice (**Fig. 2C**), as confirmed by the electrophoretic SDS-PAGE analysis of MyHC subtypes expression in the whole EDL muscle (**Fig. 3B**).

### Effects of PTN Overexpression on Muscle Vascularization in Ground, HU, and SF Mice

**Figure 4:**
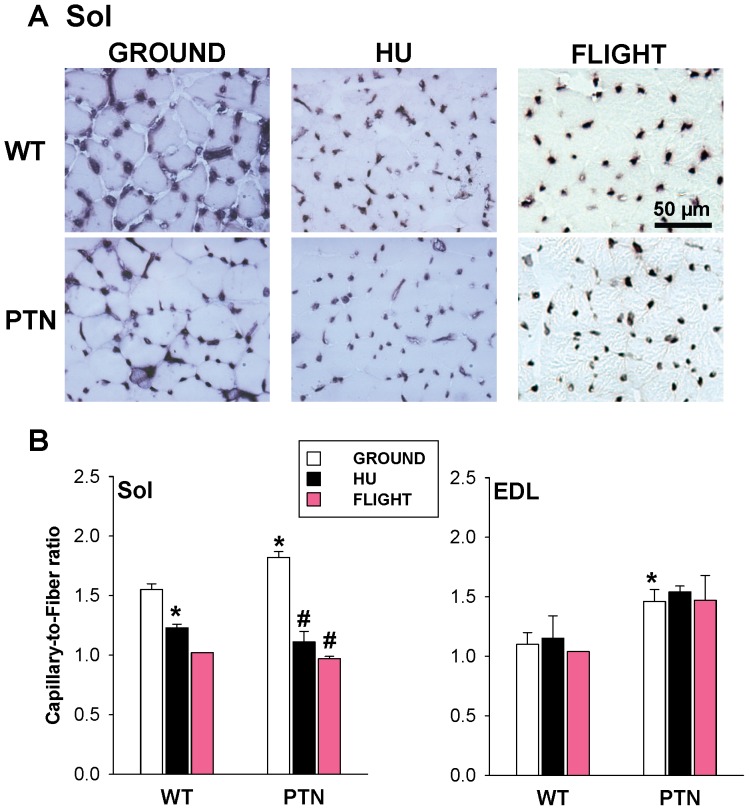
Muscle vascularization in wild-type and PTN-overexpressing mice after 14 days HU or 91 days space flight. A) Representative Sol muscle sections stained for alkaline phosphatase activity. Pictures of spaceflown samples (right column) were slightly modified using Photoshop software (Adobe) to increase contrast. B) The number of capillary was counted in Sol and EDL muscle sections stained as shown in A and normalized to the number of muscle fibers. Each bars is the mean capillary-to-fiber ratio ± SEM calculated from WT-ground (n = 6), WT-HU (n = 3), WT-spaceflown (n = 1), PTN-ground (n = 6), PTN-HU (n = 3), and PTN-spaceflown (n = 2) mice. Statistical analysis performed with two-tailed unpaired Student’s *t* test indicates significant change (*P*<0.05) versus WT-G (*) and PTN-G (#).

It is noteworthy that muscle vascularization depends on muscle activity [20]. Muscle capillary regression has been observed in rats or mice after HU [21]–[24] and in rats after SF [25]. Thus we calculated the capillary-to-fiber (C/F) ratio in Sol and EDL muscle cryosections stained for alkaline phosphatase activity (**Fig. 4A**). As expected, HU significantly reduced the C/F ratio by 21% in atrophied Sol muscle, but had no effect on the EDL muscle of WT mice (**Fig. 4B**). The C/F ratio was also greatly reduced in the SF WT mouse. In ground conditions, the over-expression of PTN induced a significant increase of C/F ratio in both Sol (+17%) and EDL (+33%) muscles, suggesting pro-angiogenic effects of the trophic factor (**Fig. 4B**). Nevertheless, PTN over-expression was unable to prevent capillary regression in Sol muscle induced by either HU or SF.

### Effects of PTN Overexpression on the Resting Conductance of Sarcolemma of Ground and HU Mice

The chloride (gCl) and potassium (gK) ion conductance of sarcolemma at rest is critically involved in the control of muscle excitability in a phenotype-dependent manner. In slow-twitch muscles the gCl is typically lower with respect to fast-twitch ones, but increases after HU either in rats or mice, in accord with the partial slow-to-fast phenotype shift [14], [17], [26], [27].

**Figure 5:**
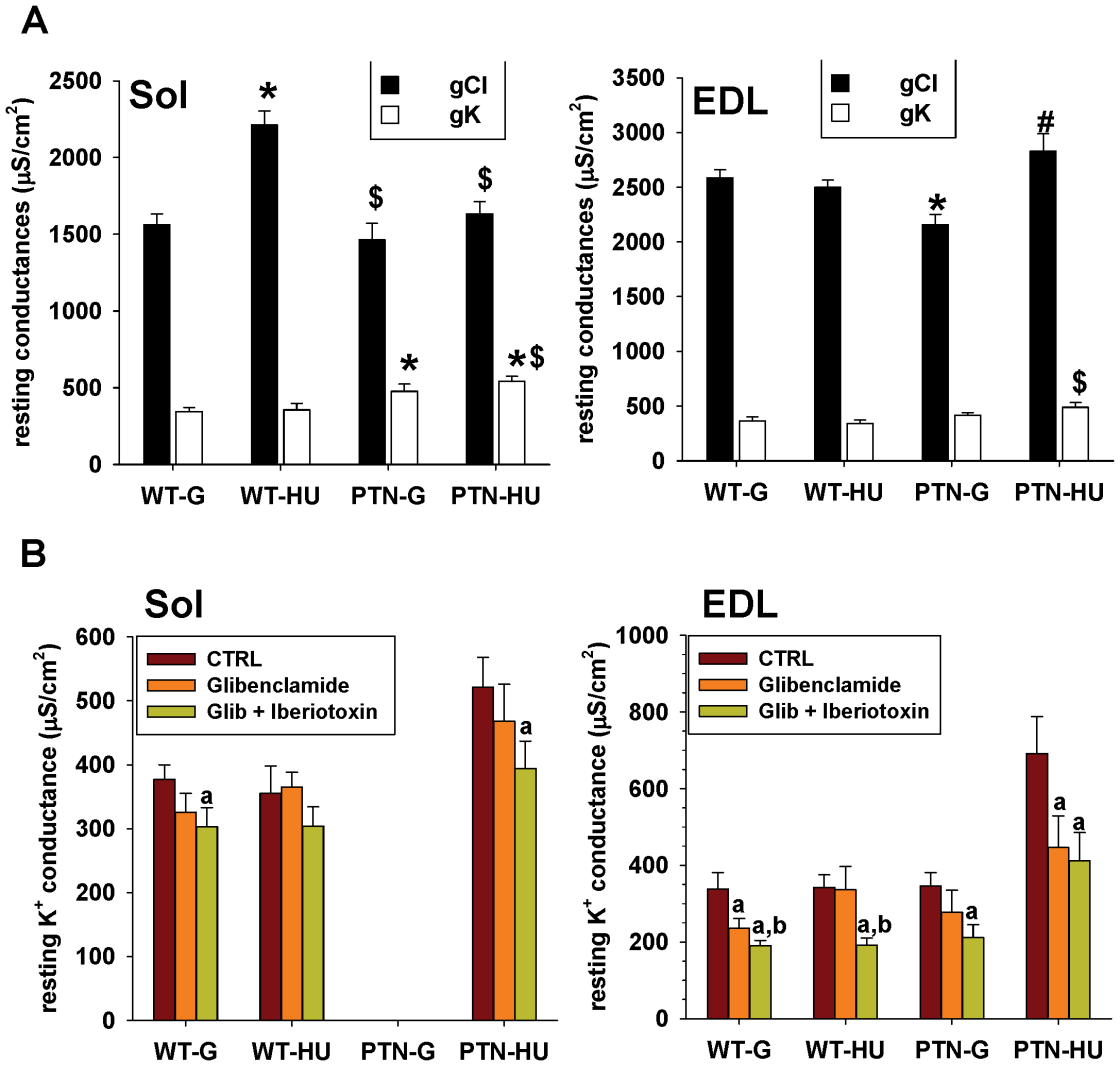
Resting potassium and chloride conductance of Sol and EDL muscle fibers of wild-type and PTN-overexpressing mice after 14 days HU. A) The chloride and potassium conductance of sarcolemma at rest were measured in current-clamp mode with the two-intracellular microelectrodes technique. Each bar is the mean conductance ± SEM calculated from 16-to-53 muscle fibers of 3-to-6 mice. Statistical analysis performed with unpaired Student’s *t* test indicates significant change (*P*<0.05) versus WT-G (*), WT-HU ($), and PTN-G (#). B) Selective inhibitors of ATP-sensitive potassium channels (50 µM glibenclamide) and large conductance, Ca^2+^-activated potassium channels (50 nM iberiotoxin) were applied in vitro to soleus muscles fibers to evaluate the contribution of these channels to the resting potassium conductance of sarcolemma. Statistical analysis performed with paired Student’s *t* test indicates significant change (*P*<0.05) versus CTRL (a) or glibenclamide (b).

A similar effect was observed in WT mice in the present study (**Fig. 5A**). In ground conditions, the gCl of Sol muscle fibers was 40% lower compared to the gCl of EDL muscle fibers but significantly increased by 42% after HU. The overexpression of PTN did not determine any change of gCl in Sol muscle in ground conditions, but prevented the increase of gCl induced by HU. No change in the gK was found in Sol muscle of WT mice after HU, as expected from previous studies. In contrast, PTN significantly increased the gK by 38% and 52% in Sol muscles of ground and HU mice.

In the EDL muscle of WT mice, HU had no significant effect on both gCl and gK (**Fig. 5A**). Surprisingly, PTN induced a significant reduction of EDL muscle gCl in ground condition, without altering the gK. After HU, both the gCl and gK increased by 13% and 43% in the EDL muscle of PTN mice.

In an attempt to identify the molecular entity responsible for the gK increases in PTN mice, we tested the effects of the ATP sensitive K^+^ channel (K_ATP_) blocker, glibenclamide [28], and of the large-conductance, calcium activated K^+^ channel (BK) blocker, iberiotoxin [29], on gK in Sol and EDL muscles. Application of glibenclamide to Sol muscle fibers reduced the gK by ∼15%, except in WT-HU mice (**Fig. 5B**). Further application of iberiotoxin produced a further ∼15% reduction of the gK in all the experimental conditions. In EDL muscles, a 15–35% reduction of gK by glibenclamide was observed in all the conditions, except in WT-HU mice (**Fig. 5B**). Iberiotoxin further decreased the gK in all the four experimental groups. These data suggest that HU abolished the contribution of K_ATP_ channels to resting K^+^ conductance in WT but not in PTN mice. Indeed, the increased gK in PTN mice after HU appeared to be mainly related to an increased activity of K_ATP_ channels.

### Effects of PTN Overexpression on Muscle Calcium ion Homeostasis in Ground and HU Mice

**Figure 6:**
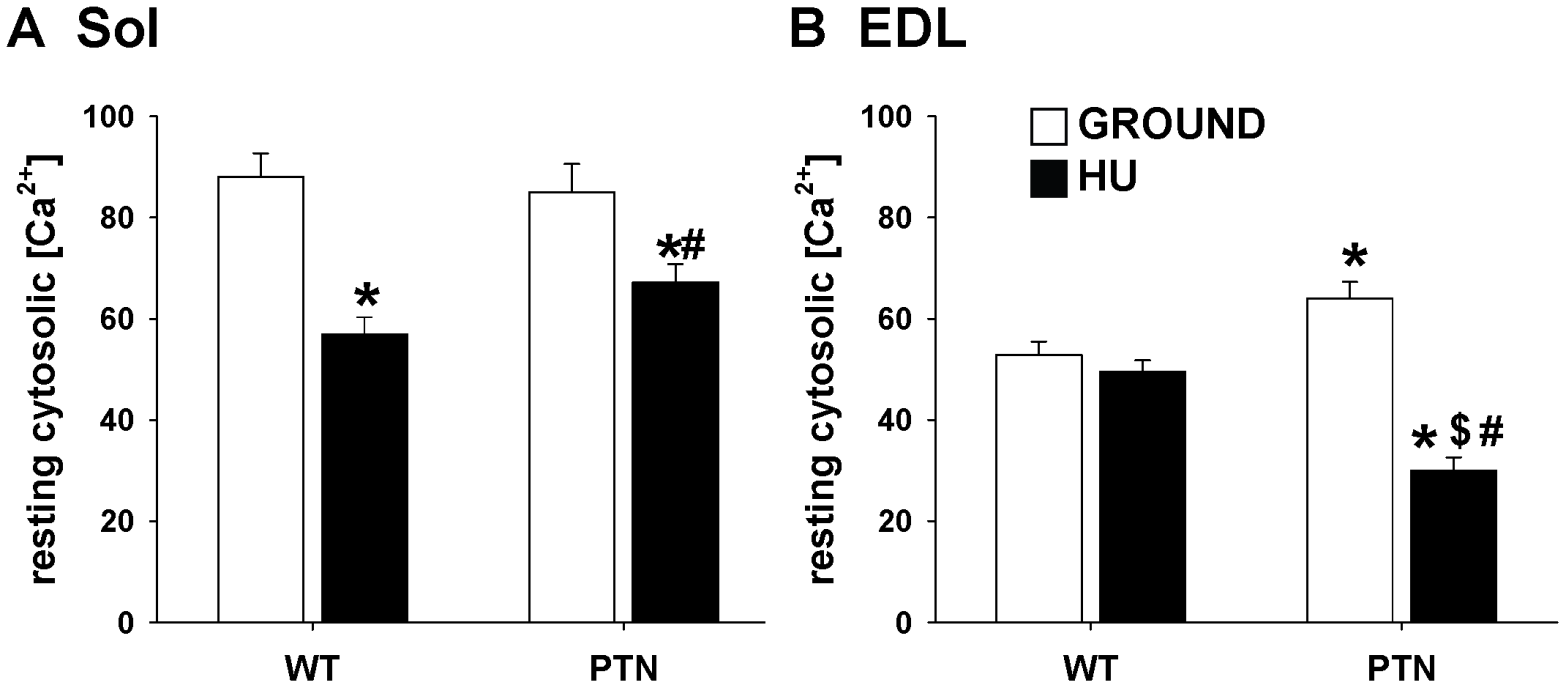
Cytosolic Ca^2+^ concentration at rest in Sol (A) and EDL (B) muscle fibers of wild-type and PTN-overexpressing mice after 14 days HU. The cytosolic Ca^2+^ concentration was determined in mechanically-dissociated muscle fibers with cytofluorescent imaging technique using the Ca^2+^ dye FURA-2. Each bar is the mean ± SEM calculated from 13-to-47 muscle fibers of 2-to-3 mice. Statistical analysis performed with unpaired Student’s *t* test indicates significant change (*P*<0.05) versus WT-G (*), WT-HU ($), and PTN-G (#).

The cytosolic calcium ion concentration at rest (restCa) depends on muscle phenotype, being higher in fibers of slow-twitch Sol muscle compared to fast-twitch EDL or gastrocnemius muscles [30]. We previously demonstrated that HU reduces restCa in Sol muscle fibers of rats and mice, in accord with the slow-to-fast shift of muscle phenotype [14], [18]. In the present study, restCa was 1.66 times higher in Sol muscle fibers compared to EDL muscle fibers of WT mice maintained in ground conditions (**Fig. 6**). As expected, restCa was significantly reduced by about 35% in WT Sol muscle fibers after HU, whereas no effect was found in EDL muscle fibers. In Sol muscle fibers, PTN over-expression did not affect restCa in ground conditions, nor the HU-dependent reduction. In EDL muscle fibers, PTN overexpression significantly increased restCa by 21%, whereas a reduction of about 50% was found after hindlimb unloading.

### Effects of PTN Overexpression on Muscle Gene Expression in Ground, HU, and SF Mice

**Figure 7:**
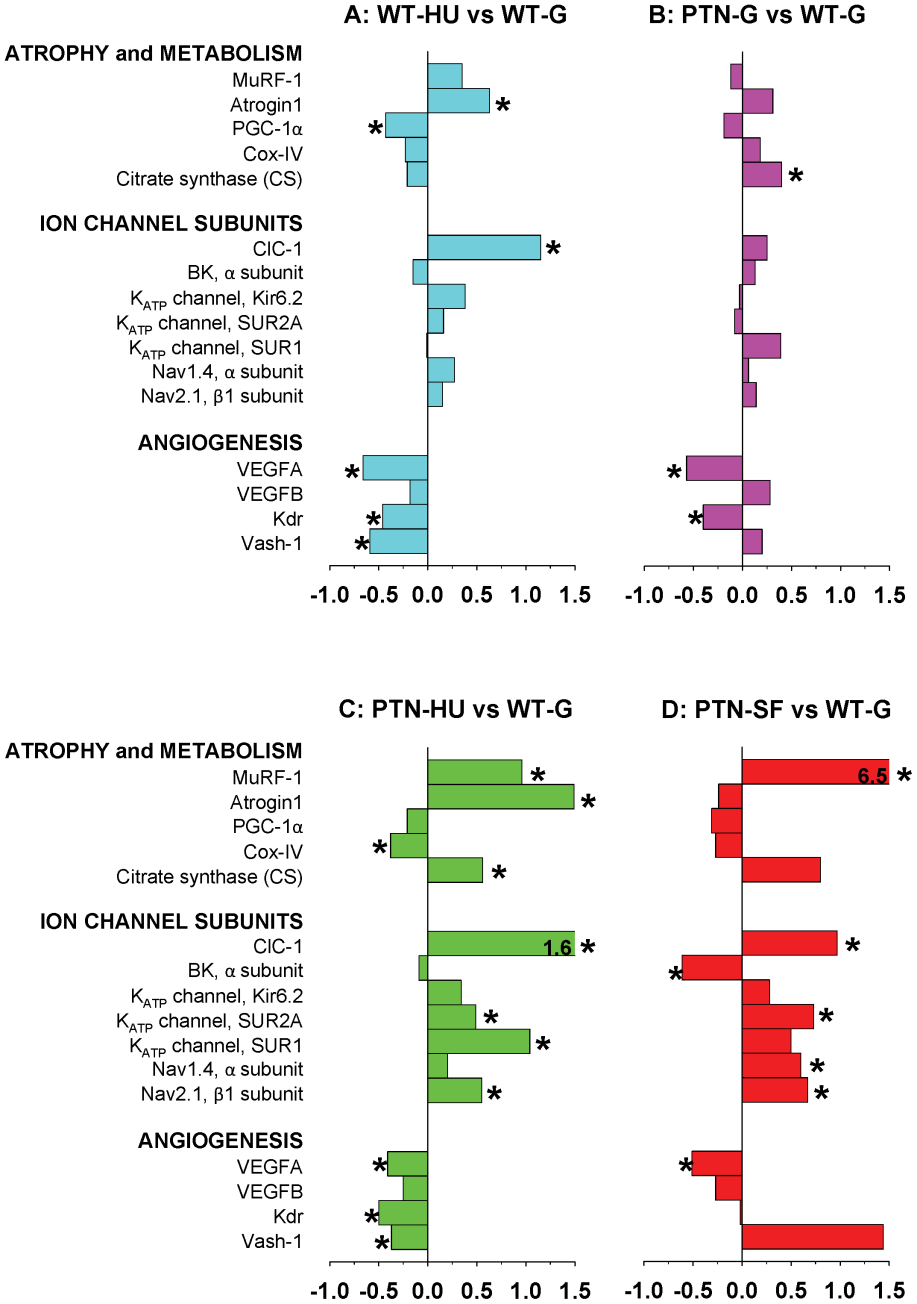
Variations in gene expression in Sol muscle induced by hindlimb-unloading and spaceflight. Transcript levels were determined by real-time PCR for selected genes, classified on the basis of the functional role of the protein they encode, and normalized for the housekeeping HPRT-1 gene. The bars indicate the fold change in gene expression between A) HU and ground conditions in WT mice, B) PTN and WT mice in ground conditions, C) PTN-HU versus WT-G mice, and D) spaceflown PTN mice versus ground WT mice. Significant fold changes, according to two-tailed unpaired Student’s *t*-test, are indicated with* (at least P<0.05).

**Figure 8:**
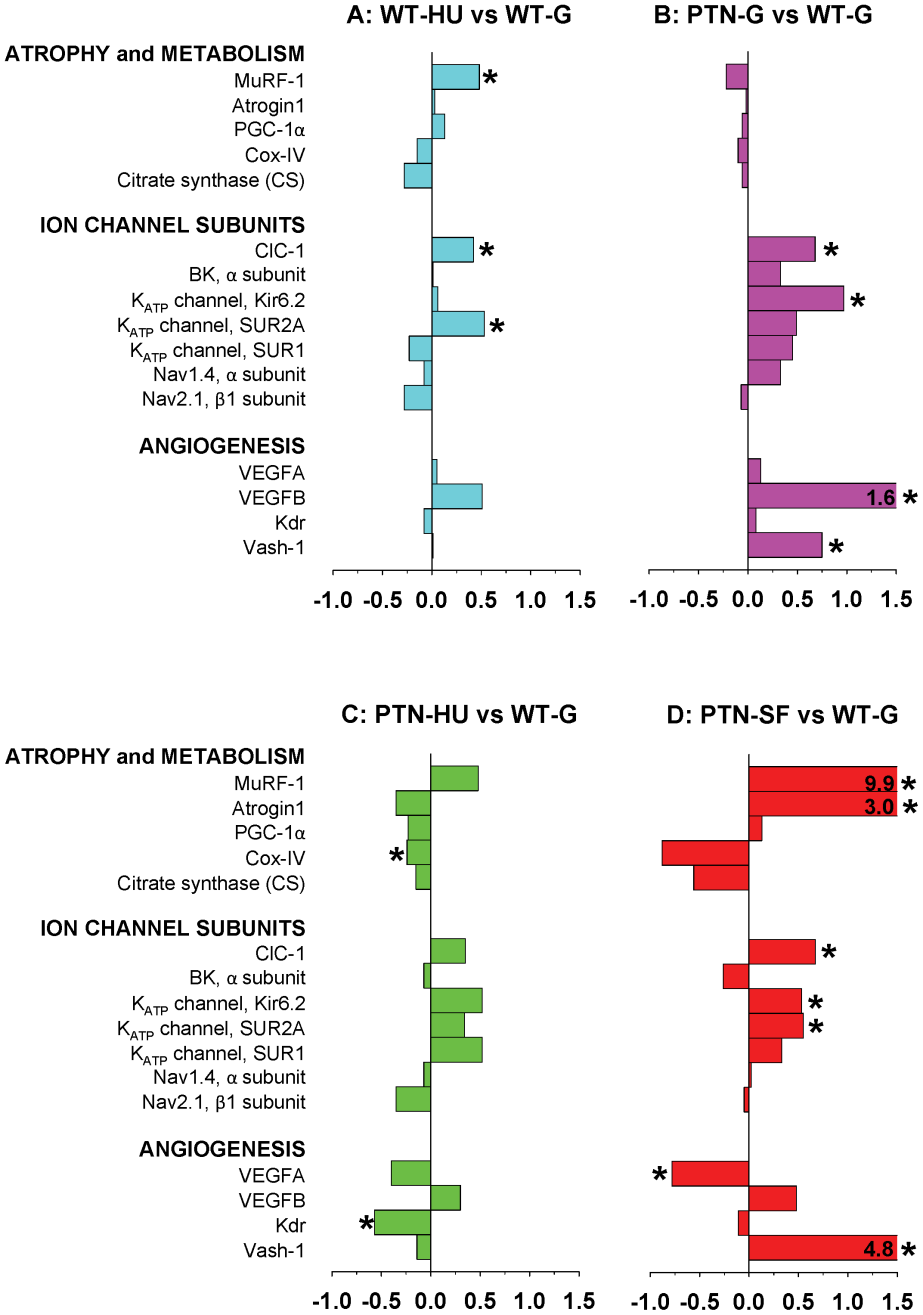
Variations in gene expression in EDL muscle induced by hindlimb-unloading and spaceflight. Transcript levels were determined by real-time PCR for selected genes, classified on the basis of the functional role of the protein they encode, and normalized for the housekeeping HPRT-1 gene. The bars indicate the fold change in gene expression between A) HU and ground conditions in WT mice, B) PTN and WT mice in ground conditions, C) PTN-HU versus WT-G mice, and D) spaceflown PTN mice versus ground WT mice. Significant fold changes, according to two-tailed unpaired Student’s *t*-test, are indicated with* (at least P<0.05).

We evaluated the adaptation changes of gene expression due to PTN overexpression in ground and actual and simulated microgravity by measuring the shifts in mRNA levels of selected genes, including genes involved in muscle atrophy and metabolism, genes encoding sarcolemma ion channels, and genes involved in angiogenesis. To investigate the effects of HU in WT mice and effects of PTN in all experimental conditions (ground, HU, and SF), we report the gene expression level ratio in Sol (**Fig. 7**) and EDL (**Fig. 8**) muscles as a comparison between A) HU and ground conditions in WT mice, B) PTN and WT mice in ground conditions, C) PTN-HU versus WT-G mice, and D) PTN-SF mice versus ground WT mice. Effects of SF on selected gene expression in Sol and EDL muscles of WT mice have been previously reported [13].

In Sol muscle of WT mice, HU induced an increased expression of the muscle-specific E3 ubiquitin ligases, atrogin-1 and MuRF-1, as expected from the atrophic process, and a reduced expression of peroxisome proliferator-activated receptor gamma coactivator-1α (PGC-1α), cycloxygenase IV (Cox-IV), and citrate synthase (CS), as expected from a shift of Sol muscle fibers toward a less oxidative phenotype (**Fig. 7A**). The observed increased expression of ClC-1 chloride channel confirms previous studies [14], and likely accounts for the increased gCl measured in HU Sol muscle. Expression changes in other ion channel subunits are less pronounced but in accord with the change in soleus muscle phenotype, with a reduced expression of BK channels, and an increased expression of K_ATP_ channel and voltage-gated sodium channel subunits. The other marked effect of HU regards the expression of angiogenesis-related genes, with a significant reduction of vascular endothelial growth factor-A (VEGFA), VEGF type 2 receptor (Kdr), and vasohibin-1 (Vash-1) expression.

In Sol muscle of ground mice, PTN overexpression had little impact on ion channel gene expression, but increased expression of Cox-IV and CS in accord with the shift of PTN Sol muscle toward a more oxidative phenotype, as suggested by changes in MyHC protein isoforms (**Fig. 7B**). Quite surprisingly, a decreased expression of VEGFA and Kdr and an increased expression of Vash-1 were found, possibly as a compensatory mechanism to pro-angiogenic effects of PTN. Nevertheless, expression of VEGFB was slightly enhanced in PTN mice. Effects of HU on gene expression in soleus muscles of PTN mice were similar to those observed in WT mice, although the changes were accentuated in the transgenic mice (**Fig. 7C**). Importantly, PTN was not able to prevent neither the HU-induced increase of atrogenes, MuRF-1 and atrogin-1, nor the reduced expression of angiogenesis-related genes. Of note is the increased expression of CS and sulfonylurea receptor 1 (SUR1) in HU-PTN mice.

The effects of SF on Sol muscles in PTN mice also recapitulated those observed in WT mice [13], characterized by an increased expression of MuRF-1, a reduction of PGC-1α, and changes in ion channel gene expression in accord with the slow-to-fast phenotype transition (**Fig. 7D**). The effects of actual microgravity in PTN mice were also comparable to those of simulated microgravity, except for the limited effect on atrogin-1 expression and the increased expression of Vash-1 after 91-days space journey.

In the EDL muscle of WT mice, HU increased the expression of MuRF-1, ClC-1, SUR2A, and VEGFB (**Fig. 8A**). In ground conditions, PTN overexpression modified gene profile in EDL muscle, by modifying expression of ion channel subunits (ClC-1, BK, Kir6.2, SUR2A, SUR1, and Nav1.4) and angiogenesis markers (VEGFB and Vash-1) (**Fig. 8B**). The effects of HU on EDL muscle of PTN mice were similar to those observed in WT mice, except for the increased expression of SUR1 and the more pronounced reduction of VEGFA and Kdr (**Fig. 8C**). As already observed for WT mice [13], analysis of EDL muscles of SF PTN mice showed changes in ion channel genes, atrogenes, and angiogenes revealing a possible activation of atrophic and fast gene programs albeit the apparent insensitivity of EDL muscle structure and function to actual microgravity (**Fig. 8D**). Noteworthy, conversely to HU, SF induced an increased expression of Vash-1 in EDL muscle as observed in the Sol muscle.

## Discussion

### Effects of Disuse on Mouse Skeletal Muscles

Our previous and current studies show that, after 14 days HU, the postural Sol muscle of mice undergoes a severe atrophy related to activation of ubiquitin-dependent proteolysis, a transition toward a less oxidative metabolism, and a vascular regression [14], [31]. Accordingly the expression of many genes encoding proteins related to atrophy, oxidative metabolism, VEGF signaling, and sarcolemma ion channels was changed after HU. Although results obtained in SF mice can be only considered as preliminary due to the limited number of available animals, they suggest that SF may share many of these effects with HU [13]. Recently a microarray data analysis of gastrocnemius muscles of mice after 12 days SF or HU suggested that only a limited number of genes may vary in the same way in both conditions [32]. The similar changes found in our study suggest that the selected genes are determinant in the adaptation of Sol muscle to disuse, whatever the model considered. On the other hand, the different conditioning durations do not allow to draw any hypothesis regarding the diverging results between the two experiments, such as the expression of the ubiquitin ligase, atrogin-1, that was up-regulated after HU but unchanged after SF.

Analysis of HU mice strongly supports that the changes in gene expression induce, or at least contribute, to the functional adaptation of Sol muscle to simulated microgravity. For instance, the increased expression of *Clcn1* gene encoding the skeletal muscle chloride ClC-1 channel is responsible for the increased sarcolemma conductance at rest in Sol muscle. Since the gCl is critical for a proper excitability and resistance to fatigue of muscles [26], [33], its alteration in microgravity conditions may affect muscle function, especially upon return to normal gravity. The reduced expression of genes involved in VEGF signaling is also likely involved in the vascular regression observed in Sol muscle, as previously suggested in HU rat experiments [24]. Importantly, we observed a similar parallelism in spaceflown mice. Interestingly, a straight relation between activation of PGC-1α and VEGFA expression has been demonstrated in skeletal muscle during hypoxia and exercise [34]–[36]. Accordingly, we observed a parallel reduction of PGC-1α and VEGFA gene expression in Sol muscle during simulated and actual microgravity. Vasohibin-1 (Vash-1) is considered as an endothelium-derived negative feedback regulator of angiogenesis [37]. Although expression of Vash-1 protein is increased in rat Sol muscle after 5 days HU [38], we observed a reduction of Vash-1 transcript after 14 days HU, which might reflect a possible feedback activation of compensatory mechanisms in the mouse. In addition, the reduction of CS gene expression in Sol muscle after 91 days SF is in agreement with the reduced CS activity measured by others in gastrocnemius muscle of mice after 12 days SF [39]. All these changes together with those in Cox-IV and ion channel subunits, are in accord with an adaptation of Sol muscle toward a less oxidative metabolism. Interestingly, in accord with the link between glycolytic metabolism and K_ATP_ channel expression [28], [40], the gene expression of K_ATP_ channel subunits was increased after HU and SF; Nevertheless, the contribution of K_ATP_ channels to the resting potassium conductance (gK) was abolished in HU muscles, most probably because this channel is also particularly sensitive to atrophy [41].

Although the EDL muscle fibers were not atrophied, an increased gene expression of ubiquitin ligases was found after HU or SF. Again, these changes are probably counterbalanced by post-transcriptional regulation and/or compensatory mechanisms, such as activation of growth pathways and stress response genes, which may protect the EDL muscle in microgravity [13]. Similarly, the modified gene expression of *Clcn1* or angiogenes after HU and spaceflight have little or no effect on the sarcolemma chloride conductance or capillary-to-fiber ratio in EDL muscle. It is worth mentioning that such compensatory mechanisms may represent druggable targets to develop efficient countermeasures against disuse-induced muscle impairment.

### Effects of PTN Overexpression on Mouse Skeletal Muscles

Albeit PTN was overexpressed in bones of transgenic mice, a number of effects were observable in Sol and EDL muscles, suggesting a paracrine action of the cytokine. In ground conditions, a shift toward a more oxidative metabolism was observed in Sol muscle of PTN mice, as evidenced by an increased number of the highly oxidative type 1 fibers, increased gene expression of SUR1, Cox-IV, and CS, and increased vascularization. PTN also greatly increases the C/F ratio in the EDL muscle. Importantly, a forced conversion of muscles toward a more oxidative metabolism have been proposed as a powerful countermeasure in various physiopathological conditions, including type 2 diabetes or muscle dystrophies [42], [43]. Thus PTN supplementation to skeletal muscles might merit further attention in such conditions.

The pro-angiogenic effect of PTN in Sol muscle is likely not related to activation of PGC-1α/VEGFA pathway, because PTN mice have a reduced gene expression of both factors. This is in agreement with previous studies showing an inhibition of VEGF signaling by PTN in cultured human umbilical vein endothelial cells [44]. Thus VEGFA, as well as Vash-1, the expression of which is increased in PTN mice, might underscore the activation of a negative feedback to pro-angiogenic effects of PTN. On the other hand, an increased expression of VEGFB was measured in Sol and EDL of PTN mice, which might contribute to the pro-angiogenic action of the trophic factor.

Despite the promotion of oxidative metabolism and angiogenesis by over-expressed PTN in ground conditions, very little protective effects of the cytokine was found in actual or simulated microgravity. The lack of prevention of vascular regression further supports the hypothesis that pro-angiogenic PTN effect and anti-angiogenic effect of microgravity are not mediated by the same signaling pathway and cannot compensate for each other. The cytokine has no effect also on the expression of ubiquitin ligases and muscle atrophy. Regarding oxidative metabolism, only the expression of CS gene was increased in Sol muscle of PTN mice after HU and SF, as it is also in ground conditions. Of note are the effects of PTN on ion channel function in Sol muscle after HU and SF. Although the gene expression of ClC-1 increases in PTN mice, as in WT mice after HU and SF, the HU-induced increase of gCl is not observed in transgenic mice suggesting that the cytokine acts at post-transcriptional level. In addition, a specific increase of gK is observed in Sol and EDL muscles of PTN mice after HU, likely due to an increased activity of K_ATP_ channels. Accordingly, the expression of K_ATP_ subunits was increased in PTN mice after HU and SF. PTN modifies also the resting Ca^2+^ concentration, especially in the EDL muscle. Altogether, these effects suggest that PTN may modulate at transcriptional and post-transcriptional levels the expression/activity of key players involved in muscle excitability and contractility.

In conclusion, the study shows that muscle response to the reduction of mechanical loading induced by hindlimb-unloading induces a number of adaptative processes, including the activation of specific gene programs. The various effects of PTN overexpression on muscle gene expression and functional parameters demonstrate the sensitivity of muscle fibers to the cytokine. We do not know how much cytokine is produced by transgenic bones and reach muscles, which represents a limitation of the study, and we cannot exclude that higher concentrations of the growth factor may exert more marked effects onto muscles. Further experiments aimed at testing effects of known PTN concentrations on skeletal muscle are thus warranted. Interestingly, preliminary results obtained in mice from the MDS mission suggest that SF may share a number of these effects with HU. Importantly, although PTN showed little benefit against HU-induced muscle disuse, the growth factor may emerge useful in various muscle diseases, because it exerts a number of synergetic actions on muscle fibers and vessels, which could enforce oxidative metabolism and ameliorate muscle performance.

## Supporting Information

Figure S1 (PDF)

Table S1 **Quantitative real-time PCR primers.**
(PDF)
