# Supplementary material for: Effects of Pleiotrophin Overexpression on Mouse Skeletal Muscles in Normal Loading and in Actual and Simulated Microgravity
Source: PLoS One. 2013 Aug 28;8(8):e72028. doi: 10.1371/journal.pone.0072028 (PMC3756024; doi:10.1371/journal.pone.0072028)
Supplement: Figure S1 — (PDF) [file pone.0072028.s001.pdf]

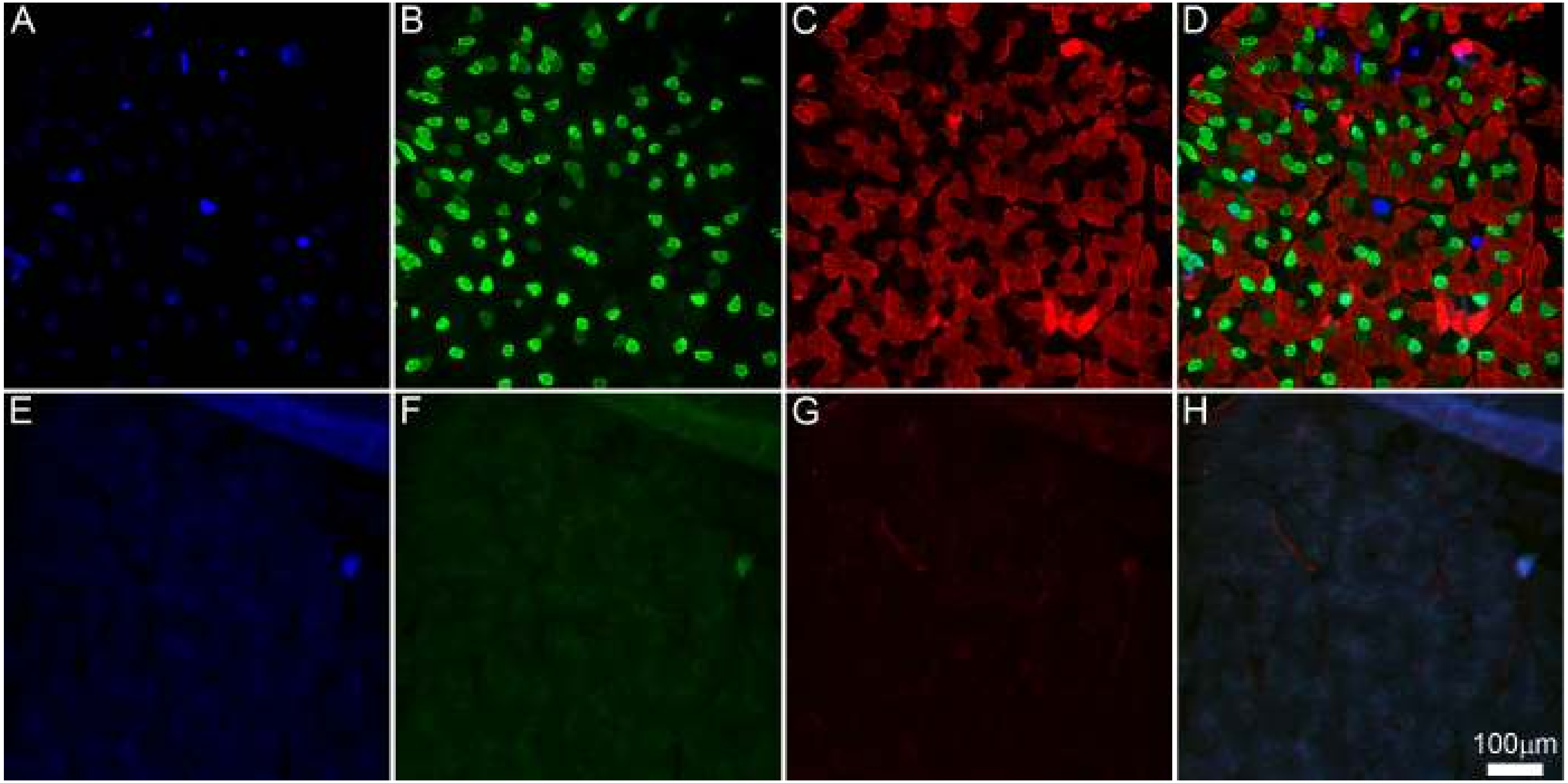

Figure S1
Muscles were incubated with BA-D5 (MyHC-slow specific, ***A***), SC71 (MyHC-2A specific, ***B***) or BF-F3 (MyHC-2B specific, ***C***) primary antibodies. A mock incubation with primary antibody solution diluent (PBS plus 0.5% BSA) was performed on serial sections in parallel, to check for background fluorescence (***E***, ***F***, ***G***). Secondary antibodies were then used as described in the materials and methods section. ***D***, merge of ***A***, ***B*** and ***C***. ***H***, merge of ***E***, ***F*** and ***G***. Increased background in ***E***, ***F***, ***G***, ***H*** is due to longer exposure time (1.5 s) compared with ***A***, ***B***, ***C*** and ***D*** (0.8 s), to allow for visualization of the fibers.

Camerino et al., Effects of pleiotrophin overexpression on mouse skeletal muscles in normal loading and in actual and simulated microgravity
