# Supplementary material for: Effects of Pleiotrophin Overexpression on Mouse Skeletal Muscles in Normal Loading and in Actual and Simulated Microgravity
Source: PLoS One. 2013 Aug 28;8(8):e72028. doi: 10.1371/journal.pone.0072028 (PMC3756024; doi:10.1371/journal.pone.0072028)
Supplement: Table S1 — Quantitative real-time PCR primers. (PDF) [file pone.0072028.s002.pdf]

TABLE S1: Quantitative real-time PCR primers

Protein	***gene***	***NCBI sequence***	***TaqMan Probe***	***forward primer (5' → 3')***	***reverse primer (3' → 5')***	***amplicon***	***efficiency***
Chloride channel 1 (ClC-1)	*Clcn1*	NM_013491.2	ATTGGCTGAGACACTTGT	TCATGCTCGGTGTCCGAAA	CAGGCGGTGCTTAGCAAGA	59	92
Ca^{2+}-activated K^{+} channel α1-subunit (BK)	*Kcnma1*	NM_010610.3	CAGAGTCCTGGTTGTGTTA	GATATCCGCCCAGACACTGACT	AGTATATTACGAGGGCACCAATGC	80	95
ATP-sensitive K+ channel (Kir6.2)	*Kcnj11*	NM_010602.2	CTGGCTCCTAGTGACCTG	CTCATCATCTACCACGTCATCGA	GTTTCTACCACGCCTTCCAAGA	116	90
ATP-sensitive K+ channel (SUR2A)	*Abcc9*	NM_021041.2	CATAGCTCATCGGGTTC	CGGATCGCACGGTCGTA	TAACCAGGTCTGCAGTCAGAATG	63	93
			Reference number from Applied Biosystems				
hypoxanthine guanine phosphoribosyl transferase 1	*Hprt*	NM_013556.2	Mm_00446968_m1			65	98
Sodium channel α-subunit (Nav1.4)	*Scn4a*	NM_133199.2	Mm_01258366_m1			86	94
Sodium channel β1-subunit (Nav2.1)	*Scn1b*	NM_011322.2	Mm_00441210_m1			86	97
ATP-sensitive K+ channel, SUR1	*Abcc8*	NM_011510.3	Mm_01701349_m1			82	99
Atrogin1 E3 ligase	*Fbxo32*	NM_026346.3	Mm_00624629_m1			72	91
MuRF-1 E3 ligase	*Trim63*	NM_001039048.2	Mm_01185221_m1			57	89
PGC-1α	*Ppargc1a*	NM_008904.2	Mm_01208835_m1			68	91
cytochrome c oxidase subunit IV (COXIV)	*Cox4i1*	NM_009941.2	Mm_01250094_m1			106	90
citrate synthase	*Cs*	NM_026444.3	Mm_00466043_m1			57	89
vascular endothelial growth factor A	*Vegfa*	NM_001025250.3<br>NM_001110266.1<br>NM_001110267.1<br>NM_009505.4	Mm_01281449_m1			81	92
vascular endothelial growth factor B	*Vegfb*	NM_011697.3<br>NM_001185164.1	Mm_00442102_m1			93	95
kinase insert domain protein receptor	*Kdr*	NM_010612.2	Mm_01222421_m1			64	97
vasohibin 1	*Vash1*	NM_177354.4	Mm_00616592_m1			90	91
